# Exploring healthcare providers’ mental models of the infection prevention “patient zone” - a concept mapping study

**DOI:** 10.1186/s13756-019-0593-4

**Published:** 2019-08-14

**Authors:** Jasmina Bogdanovic, Serge Petralito, Simone Passerini, Hugo Sax, Tanja Manser, Lauren Clack

**Affiliations:** 1 Division of Infectious Diseases and Hospital Epidemiology, University Hospital Zurich and University of Zurich, Rämistrasse 100, CH-8091 Zurich, Switzerland; 2 Institute of Nursing Science, University Basel, Basel, Switzerland; 3 FHNW School of Applied Psychology, University of Applied Sciences and Arts Northwestern, Northwestern, Switzerland

**Keywords:** Patient zone, Concept mapping, Mental models, Hand hygiene, Infection prevention and control, Card-sorting, Qualitative research, Interview, Transmission

## Abstract

**Background:**

Pathogen transmission plays a major role in the development of healthcare-associated infections. The “patient zone” concept developed as part of the World Health Organization’s “Five moments of hand hygiene” aims to distinguish surfaces primarily contaminated by flora of a single patient, i.e. *inside* the patient zone, from those *outside* the patient zone containing foreign and potentially harmful microorganisms. Discrepancies in healthcare provider (HCP) internal conceptual representations (i.e. mental models) of the patient zone may lead to missed infection prevention measures that could result in patient harm. We explored HCPs’ mental models of the patient zone that shape how they interact with the work environment.

**Methods:**

We conducted individual concept mapping interviews supported by a card-sorting technique to examine HCPs’ mental models of the patient zone and compared these to IPC expert models. Ten participants (five nurses, five physicians) without IPC specialization and two IPC experts provided definitions of the patient zone and allocated 32 items to “inside” or “outside” the patient zone while verbalizing their thought processes. We calculated similarity as percent agreement among participants and accuracy as percent allocated consistently with expert consensus. A content analysis of interview recordings served to identify mental models underlying the allocation decisions.

**Results:**

Our study revealed limited similarity among participants, with seven of 32 items allocated consistently among all participants. Overall, 68% of items were sorted accurately according to expert consensus. Identified mental models were categorized as follows: “Patient contact”, the patient zone defined according to objects having patient contact; “Sectors”, the patient zone as a defined physical space; “Disinfection”, the patient zone deduced based on need to disinfect hands and objects; and “Context-dependency”, the patient zone defined depending on the context of an object’s use.

**Conclusions:**

Our study revealed ambiguity surrounding the patient zone concept as evidenced by low similarity between participants and important discrepancies between participant and expert mental models. Such ambiguity may lead to inconsistent application of the patient zone concept and represents a patient safety risk. Initiatives to improve understanding and application of the patient zone concept should focus on establishing consistent, theoretically founded mental models.

## Background

Preventing transmission of infectious agents is a priority for preventing healthcare associated infections (HAIs). To this end, the “patient zone” concept was originally introduced in 2007 as part of the “Five Moments for Hand Hygiene” to guide infection prevention and control (IPC) efforts and to anchor specific indications for hand hygiene by distinguishing the “patient zone” from the overall “healthcare zone” [1]. Rooted in the 2006 evidence-based model for hand transmission during patient care [2], the patient zone is defined in the landmark 2007 “My Five Moments for Hand Hygiene” paper as follows: “The patient zone contains the patient X and his/her immediate surroundings. This typically includes the intact skin of the patient and all inanimate surfaces that are touched by or in direct physical contact with the patient such as the bed rails, bedside table, bed linen and infusion tubing and other medical equipment. It further contains surfaces frequently touched by healthcare workers while caring for the patient such as monitors, knobs and buttons, and other ‘high frequency’ touch surfaces within the patient zone [1].” The healthcare zone, equivalent to “outside” the patient zone, in contrast, contains all surfaces outside the patient zone, and is considered to be contaminated with microorganisms that are foreign and potentially harmful to the patient. Two indications for hand hygiene are accordingly anchored upon “entry” (i.e. before touching the first surface inside the patient zone or before touching the patient) and “exit” of the patient zone (i.e. after touching the last surface inside the patient zone and proceeding to the healthcare zone) to prevent the cross transmission of microorganisms between zones that could harm patients [2]. The patient zone concept also has implications beyond hand hygiene to include disinfection of objects and surfaces to prevent transmission of microorganisms between healthcare and patient zones [3].

The “patient zone” concept was designed to be applicable in all healthcare settings and adaptable to setting-specific needs to facilitate compliance with hand hygiene across settings. The patient zone is also at the heart of direct hand hygiene observation methods [4] and used by IPC professionals to assess and compare rates of hand hygiene across the world. It is unclear, however, to which extent the patient zone concept and its practical implications are understood or used by individuals who provide frontline patient care. Success of the patient zone concept relies on healthcare providers (HCPs) who work together having correct and shared *mental models* [5]. Mental models have been defined as internal conceptual representations of the world around us, including the beliefs, values, and assumptions we hold that shape the way we behave and interact with our environment [6–9]. Specific to the patient zone, shared mental models entail a common and correct understanding about which items belong to the patient zone and should therefore be protected from foreign and potentially harmful microorganisms. Both aspects, accuracy and similarity, of mental models are essential to ensuring system safety with respect to the patient zone – where *accuracy* ensures a correct and clinically meaningful separation of microorganisms and *similarity* ensures consistent application across individuals. Discrepancies in HCP’s mental models of the patient zone may lead to unintentional contamination of the patient’s direct environment and subsequent incorrect application of IPC measures that could result in patient harm [5, 10]. Studies reporting hand hygiene compliance as low as (35–65%) after contact with objects in the patient zone suggest that there is indeed high potential for unintended transmission of microorganisms [11, 12], and this low compliance may be, in part, related to incongruent mental models of the patient zone. To our knowledge there are no scientific studies addressing HCPs’ mental models of the patient zone. Concept mapping has been proposed as a suitable method to study mental models [9, 13–15]. In the current study, we aim first to elicit and assess the similarity and accuracy of HCPs’ item allocations to inside or outside the patient zone and then to qualitatively assess the mental models behind these sorting decisions.

## Methods

### Study design and aim

We conducted a concept mapping study to explore HCPs’ mental models of the patient zone. Participating HCPs without specialized training in IPC (“participants”) completed a card-sorting task together with verbal think-aloud protocol, in which they allocated items from the healthcare environment to “inside” or “outside” the patient zone. Participant allocations of items were subsequently compared with expert consensus allocations, established by one IPC nurse and one infectious diseases physician (“IPC-experts”), which served as our ground truth. We assessed both the similarity and accuracy of HCP’s mental models. *Similarity* is defined as the extent of agreement among participants and *accuracy* as the proportion of participants who sorted items according to IPC-expert consensus. Furthermore, the qualitative data from the think-aloud protocol served as the basis for identifying mental models informing HCPs’ allocation decisions.

### Participants and setting

We included a convenience sample of HCPs who were willing to participate. We recruited nurse and physician participants without specialization in infection prevention from a general medical ward. Inclusion of participants continued until theoretical saturation was achieved, that is until no new ideas were emerging from data collection.

### Procedure

**Fig. 1:**
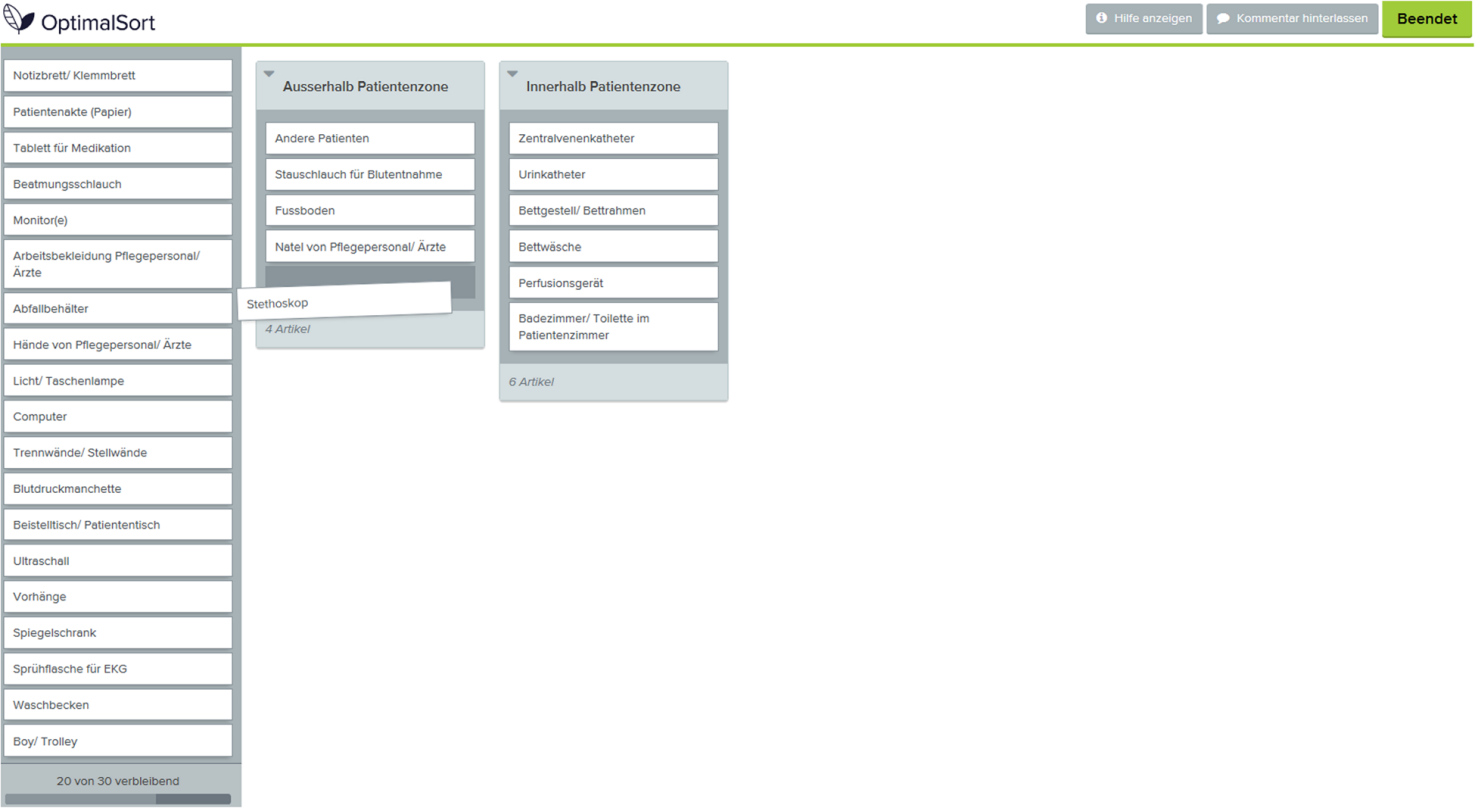
Participants used the online card-sorting tool to attribute items to "inside patient zone" or "outside patient zone

We conducted the card-sorting sessions as semi-structured interviews taking place between January and February 2018. First, participants provided a definition of the patient zone in his or her own words. Then, they filled out a short questionnaire with their demographics (age, gender, profession, professional experience) and performed the card-sorting activity on a computer using the online tool OptimalSort (Optimal Workshop, Wellington NZ). Figure 1 shows a screenshot of the online-tool. Participants were provided with a scenario set in a two-patient room on a general ward and a set of cards with 32 items from the care environment. The list of items used for the cards was generated based on an observational study [10, 16]. The researcher instructed the participants to allocate the cards into two pre-defined categories “inside patient zone” or “outside patient zone”. Although, some items were difficult to sort, participants were asked to make a decision and declare why they would rather allocate the item to the particular zone. Participants were also instructed to “think-aloud” throughout the card-sorting activity, verbalizing their thought process [17]. After the card-sorting activity, participants gave a subjective rating of their own knowledge about the patient zone concept on a five-point Likert scale. One researcher with training in psychology and familiar with the infection prevention context (JB) performed data collection. All sessions were video recorded to attribute the spoken word from the think-aloud to what was happening in the card-sorting process.

### Analysis

To assess participants’ demographics as well as the similarity and accuracy of allocations, we exported raw data from the online-tool. We assessed similarity of participant mental models by calculating percent agreement among and between professional groups for each sorted item. Accuracy was calculated as the proportion of participants allocating items consistent with both IPC experts.

Furthermore, we performed a content analysis of the concept mapping interviews using an excel spreadsheet where we captured profession, participant code, participants’ definitions of the patient zone, sorting allocation (inside vs. outside patient zone), and utterances describing reasons for allocating items. Utterances describing reasons for allocating items were inductively coded into different categories. These codes subsequently served as a basis for identifying and quantifying different mental models about the patient zone. Mental models were further analyzed, first, to determine how frequently they accompanied correct versus incorrect item allocations and, ultimately, to assess the extent to which each mental model represents a useful heuristic for decisions about which items belong to the patient zone.

## Results

Overall, 10 non-IPC-expert participants joined the study. These included five nurses and five physicians from the general medical ward. Five participants were female, with an average age of 32.25 years. Participating physicians reported to have no (*n* = 2) or slight (*n* = 3) knowledge of the patient zone concept. In contrast, participating nurses reported to have average (*n* = 4) or good (*n* = 1) knowledge. Furthermore, only one participant reported having received information or training about the patient zone. Two IPC experts from the Division of Infectious Diseases and Hospital Epidemiology, one physician and one nurse, participated to establish expert consensus.

### Accuracy

Expert consensus allocations, taken as ground truth to calculate accuracy, are shown in Table 1. Experts agreed upon all items except “curtains” and “partition walls”, which were therefore excluded from further accuracy calculations. A total of 10 participants (five nurses, five physicians) allocated 32 objects to categories of “inside” or “outside” the patient zone, resulting in 320 total allocations, and 300 after excluding curtains and partition walls. Of these, 204 were allocated consistently with expert consensus, resulting in a 68% overall accuracy. The accuracy of allocations for each individual item and professional group is shown in Table 2. The vast majority of allocation errors (87 out of 96 incorrect allocations, 83.5%) concerned objects that belong outside but were incorrectly allocated to inside the patient zone, whereas only eight errors occurred when allocating objects that belong inside the patient zone.

**Table 1:** IPC-expert consensus: allocation of items to zones

Inside Patient Zone	Mixed	Outside Patient Zone
Bedframe	Curtains	Bathroom in patient room
Bedsheets	Partition wall	Blood pressure cuff
Bedside table		Clipboard
Central Venous Catheter		Computer
Fixed telephone in patient room		Conductive gel bottle (for ECG)
Infusion pump		Floor
Monitor		Healthcare provider badge
Urinary Catheter		Healthcare provider hands
		Healthcare provider private mobile phone
		Healthcare provider professional attire
		Medication tray
		Mirrored Cabinet
		Another patient
		Paper patient records
		Pens
		Physicians’ pocket lamp
		Sink
		Stethoscope
		Tourniquet
		Trolley
		Ultrasound
		Waste bin

*ECG* electrocardiography, *IPC* Infection prevention and control

**Table 2:** Accuracy of non-IPC-expert participants’ item allocations

Inside Patient Zone^a^	Overall (%)	Nurses (%)	Physicians (%)
Bedframe^a^	100	100	100
Bedsheets^a^	100	100	100
Central Venous Catheter^a^	100	100	100
Urinary Catheter^a^	100	100	100
Bedside table	90	100	80
Fixed telephone in patient room	90	100	80
Monitor	90	100	80
Infusion pump	60	60	60
Outside Patient Zone^a^	Overall	Nurses	Physicians
Computer^a^	100	100	100
Healthcare provider private mobile phone^a^	100	100	100
Paper patient records^a^	100	100	100
Conductive gel bottle (for ECG)	90	100	80
Healthcare provider badge	90	80	100
Pens	90	80	100
Clipboard	80	80	80
Physicians’ pocket lamp	70	100	40
Trolley	70	80	60
Healthcare provider professional attire	60	60	60
Mirrored Cabinet	50	40	60
Other patient	50	60	40
Waste bin	50	20	80
Floor	40	20	60
Medication tray	40	60	20
Stethoscope	40	60	20
Tourniquet	40	60	20
Ultrasound	40	60	20
Blood pressure cuff	30	40	20
Healthcare provider hands	30	40	20
Sink	30	40	20
Bathroom in patient room	10	0	20

Legend: Numbers show the percent of participants that correctly allocated items consistent with IPC-expert consensus allocations. Items marked with ^a^ achieved 100% agreement. Entries are sorted from highest to lowest accuracy by group. Two items for which IPC-expert consensus was not achieved, namely partition walls and curtains, are not included in this figure. *ECG* electrocardiography, *IPC* Infection prevention and control

### Similarity

The similarity in allocation of items among non-IPC-expert participants is depicted in Table 3. Seven out of thirty items achieved a 100% agreement, meaning all 10 participants agreed on the items’ allocation to in- or outside the patient zone. The same seven items achieved a 100% agreement between participants and IPC-experts (Table 2).

**Table 3:** Similarity in allocation of items to zones for all 10 non-IPC-experts

Majority Inside Patient Zone (%)	Mixed (50% inside, 50% outside)	Majority Outside Patient Zone (%)
Bedframe (100)^a^	Waste bin	Computer (100)^a^
Bedsheets (100)^a^	Another patient	HCP private mobile phone (100)^a^
Urinary Catheter (100)^a^	Mirrored Cabinet	Paper patient records (100)^a^
Central Venous Catheter (100)^a^	Partition wall	Healthcare provider badge (90)^a^
Bathroom in patient room (90)		Conductive gel bottle (for ECG) (90)^a^
Bedside Table (90)^a^		Pens (90)^a^
Fixed phone in patient room (90)^a^		Clipboard (80)^a^
Monitor (90)^a^		Trolley (70)^a^
Blood pressure cuff (70)		Physicians’ pocket lamp (70)^a^
HCP hands (70)		HCP professional attire (60)^a^
Curtains (70)		
Sink (70)		
Floor (60)		
Infusion pump (60)^a^		
Tourniquet (60)		
Stethoscope (60)		
Medication tray (60)		
Ultrasound (60)		

Legend: Items designated with ^a^ were in agreement with expert consensus; *IPC* infection prevention and control, *ECG* electrocardiography, *HCP* healthcare provider

### Mental models behind card sorting decisions

Our content analysis resulted in the identification of multiple mental models underlying participants’ allocation decisions. The identified mental models were grouped into the following four categories: *Patient contact*; *Sectors*; *Disinfection*; and *Context-dependency*. The identified mental models often revealed ambiguity and inconsistent beliefs surrounding the patient zone concept, as described in the following sections and summarized in Table 4. It is important to note that one allocation of an item could be informed by multiple mental models and that these mental models are not mutually exclusive.

**Table 4:** Mental models informing item allocation decisions

Mental model	Description	Example of Correct allocation	Example of Incorrect allocation	Shortcoming
Patient contact	An object belongs inside the patient zone if it comes into contact with the patient.	“*[The computer is] only in the room for medical rounds. It has contact only with my hands.*” [Computer, correctly allocated outside]	“*As soon as there is patient contact, the clothing is contaminated with the patient’s flora.*” [HCP professional attire, incorrectly allocated inside]	This mental model falls short when it comes to mobile objects that have contact with multiple patients.
Sectors	The patient zone is a geographic zone defined by proximity to the patient or equivalent to the perimeter of the patient’s room. Inside the patient room is equivalent to inside the patient zone.	“*The trolley is mostly located outside the patient room.*” [Trolley, correctly located outside]	“*Every place that the patient is using or staying at is patient zone. Also in a two-bed patient room.*”[Bathroom, incorrectly allocated inside]	This mental model falls short when it comes to rooms with multiple patients. Incorrectly assumes that items change zone attribution, whereas experts describe patient zone attribution as a fixed characteristic.
Disinfection	Version A: The item belongs inside the patient zone if it needs to be disinfected. Version B: The item belongs outside the patient zone if it needs to be disinfected.	“*[Infusion pump is outside the patient zone] because it always needs to be disinfected.*” [Infusion pump, correctly allocated outside]	“*[They are inside the patient zone, because] hands have to be disinfected whenever you go to a patient.*” [HCP hands, incorrectly allocated inside]	The 5-moment concept patient zone is intended to inform whether an item needs to be disinfected and not the other way around. The deduction can be based on incorrect behavior (e.g. not disinfecting an item between patients).
Context-dependency	An item can be inside or outside the patient zone, depending on the context.	–	“*Depending on the situation, they [the hands] are inside the zone when the HCP is doing intervention on the patient.*” [HCP hands, incorrectly allocated inside]	Incorrectly assumes that items change zone attribution, whereas the patient zone is actually a fixed attribution.

*HCP* healthcare provider

***Patient contact*** was the most frequently occurring mental model (*n* = 103). This mental model is informed by the unreliable assumption that an item automatically belongs inside the patient zone if the item comes into contact with the patient or outside the patient zone if there is no contact. This mental model served incorrect and correct decisions alike. Whereas experts agreed that items such has HCPs’ hands and professional attire belonged to outside the patient zone, participants diverged from experts and described these items as belonging inside the patient zone, describing, for example, “*This is the part [of the HCP’s hand] that comes into contact with the patient.*” (*Physician 3*). Similarly, a nurse stated, “*As soon as there is patient contact, the [HCP professional attire] clothing is contaminated with the patient’s flora.*” (*Nurse 1*). In contrast, this mental model led to correct allocations of items that never came into patient contact to outside the patient zone. For example, a physician correctly described, “*[The computer is] only in the room for medical rounds. It has contact only with my hands (Physician 4).*” The mental model that the patient zone is defined according to whether items come into patient contact fails to account for mobile objects that come into contact with multiple patients (e.g. HCP hands, stethoscope). Based on the “patient contact” mental model, such mobile items are perceived as belonging inside the patient zone, when in fact they have potential to become contaminated with the flora of more than a single patient and therefore belong outside the patient zone.

***Sectors*** was the second most frequently occurring mental model (*n* = 83). Participants with this mental model described the patient zone as an unmoving, designated area (i.e. sector) that is equivalent to the perimeter of the patient room or a certain proximity (e.g. 2 m) to the patient. This mental model is driven by the perception that the patient zone is a stable sector and that items can be in- or outside the patient zone depending on their physical location with respect to this sector. Participants described, for example, that the stethoscope belongs to the inside patient zone when it is being used on a patient, or outside when it is being carried outside the patient room. In contrast, experts viewed patient zone attribution as a fixed characteristic of items that did not change depending on their physical location. Most frequently, participants with this mental model defined the patient zone as simply equivalent to the entire patient room without specifying the implications for rooms shared by multiple patients. Although this mental model also led to both incorrect and correct allocation decisions, it is based on the misunderstanding that the patient zone is always a stable zone into which objects can enter or exit. Although equating the patient zone to the perimeter of the patient room could be an attractive simplification, experts noted that this fails to account for rooms with multiple patients and shared spaces that belong outside the patient zone.

***Disinfection*** was the third most frequently occurring mental model (*n* = 36). Interestingly, participants seemed to deduce whether an item belonged inside or outside of the patient zone depending on whether they believed that the item needed to be disinfected. In contrast, experts approached this situation differently, using the fixed attribution of items to inside or outside the patient zone to describe the need for disinfection. As all other mental models, *Disinfection* led to both incorrect and correct allocations. In contrast to other mental models, the category *Disinfection* did not always follow the same coherent logic when making allocation decisions. Sometimes the need for disinfection served as a reason for allocating an item to inside the patient zone, in other cases it was the opposite. One participant, whose allocation of HCPs’ hands to inside the patient zone diverged from expert consensus, stated that the HCPs’ hands are inside the patient zone “*Because hands have to be disinfected whenever you go to a patient.*” (*Physician 1*). Here the participant correctly identified the practical implication, that HCPs’ hands should be disinfected prior to patient contact, but incorrectly deduced to which zone the HCPs’ hands belong when considering that experts allocated HCPs’ hands to outside the patient zone. Physician 1 correctly allocated the pocket lamp to outside the patient zone, stating, again correctly, that, “*These things always need to be cleaned between patients.*” Of note, this participant used the same argument for correctly allocating the infusion pump to inside the patient zone: “*Because it always needs to be disinfected.*” The occurrence of this mental demonstrates that HCPs acknowledge the link between the patient zone concept and indications for hand and object disinfection, yet gaps appear in the concretization of this mental model.

Another mental model worth noting was **Context-dependency** (*n* = 20). This mental model concerned items, which, according to the participant, could be either inside or outside the patient zone, depending on the context. For example, participants described that mobile objects, such as tourniquets and stethoscopes, belong to inside the patient zone when they are in use, but outside the patient zone when they are not. This mental model often frequently coincided with the *Sectors* mental model, where an object’s status as inside or outside the patient zone was perceived to change depending on whether it was in- or outside of the patient room. “*Depending on the situation, they [the HCP’s hands] are inside the zone when the HCP is caring for the patient.*” (*Nurse 5*). This mental model was most often detected when discussing mobile items that tend to move between healthcare spaces. Again, this mental model, that an item’s status as inside or outside the patient zone is dependent on the context, diverges from expert explanations of the patient zone attribution as a fixed, unchanging characteristic of items.

## Discussion

Our study revealed significant ambiguity surrounding the patient zone concept. This was evidenced by low similarity in item allocation between participants and important discrepancies between the mental models of experts and our sample of nurses and physicians from a general medical ward, which led to low accuracy in item allocation*.* Such ambiguity may lead to inconsistent application of the patient zone concept in daily practice and represents a potential patient safety risk on multiple levels. First, limited accuracy reveals that participating HCPs diverged from experts in their allocation of items to patient zones, suggesting that a correct and clinically meaningful separation of microorganisms may not always be achieved. Second, limited similarity among individual HCPs suggests that the understanding and application of the patient zone concept were not consistent. Such discrepancies in HCPs’ item allocations may lead to unintentional contamination of the patient’s direct environment, which could result in patient harm. Our participants’ mental models, which informed their allocation of items to inside or outside the patient zone, were composed of intuition-driven heuristics. Although these heuristics made intuitive sense to participants, they were unable to accurately capture the central logic of the patient zone concept as it was conceived and implemented by experts. Such misunderstandings are comprehensible, given that only one of our ten participants reported receiving formal training about the patient zone. Ultimately, the identified discrepancy between participant and expert understandings of the patient zone is a primary finding of our study, which causes us to question the intuitive accessibility of the patient zone as a concept intended to facilitate IPC application of measures.

Only seven of 32 items were allocated with 100% agreement among all participants and experts. These included objects that are in frequent contact and remain with the patient throughout the duration of the hospital stay (e.g. bedframe, bedsheets) and were therefore assigned to inside patient zone. Similarly, objects that are unlikely to come into contact with the patient (computer, HCP’s personal mobile phone, paper patient records) were also consistently assigned to outside the patient zone. This finding is consistent with **Patient contact** being the most frequently occurring mental model. The items that presented the greatest difficulty for participants to allocate were mobile items that move between different patients and the larger healthcare environment. This is further highlighted by the fact that the vast majority of allocations that diverged from expert consensus concerned objects that belong outside but were incorrectly allocated to inside the patient zone. Due to the mobile nature of these items, they have the highest risk to transmit potentially harmful microorganisms and therefore represent the biggest categorical hazards for patient safety.

Our qualitative analysis of the beliefs and internal conceptual representations underlying HCPs’ allocation decisions led to the identification of numerous mental models. The two most frequently occurring mental models fell under the categories of **Patient contact** and **Sectors**. Both of these consider the patient zone as a spatial concept based on which participants deduced whether an item belonged to the patient zone depending on contact with or proximity to the patient. We suspect that our participants’ mental models of “zones” were informed by experience with zones encountered in everyday life. These may include speed limit zones, transportation fare zones, time zones, which are typically statically defined geographic regions, through which people and items can travel and temporarily be located within, and then exit. We surmise that these mental models are further propagated by frequently employed language such as “entering” and “exiting” the patient zone – terms that are regularly employed within the IPC domain to anchor hand hygiene indications, which also promote the perception that the patient zone is a geographic sector that can be entered and exited. This is however not exclusively the case with the patient zone, in which IPC experts view attribution to inside or outside the patient zone is an un-changing characteristic of items. While it is true that many items that do come into contact with the patient and are in the patient’s proximity *also* belong to the patient, this heuristic is not true for all objects, such as stethoscopes, HCPs’ professional attire, and HCPs’ hands, which all come into contact with multiple patients and are therefore part of the outside patient zone. Additionally, the belief that the patient zone is equivalent to the patients’ room is not adequate for rooms with multiple patients and shared spaces likely to be contaminated by foreign microorganisms. Hence, the spatial heuristic would need to be further elaborated by including additional factors such as potential for contamination with different patients’ flora.

Faced with ambiguity when allocating mobile objects to a single zone, HCPs often described **Context-dependency**, stating that an item can be either inside or outside the patient zone, depending on the context (e.g. an item’s location). This further displays a common perception among participants that diverges from the expert opinion that patient zone attribution is a fixed characteristic. Even on an individual level, participants often had difficulties to remain consistent in their allocations when it came to movable objects, describing, for example, the tourniquet as inside and stethoscope as outside, although both devices play similar roles with respect to the patient zone. In such cases we were able to observe cognitive dissonance in participants, whereby they showed inconsistencies in sorting items according to the justifications they had previously cited [18]. In contrast, there was no ambiguity regarding items that remain in direct contact with the patient during the entire hospital stay such as CVC- or urinary catheters, bedframes and bedsheets, as shown by the 100% similarity of participant allocations. Other objects that also often stay with the patient for the duration of hospitalization (e.g. monitor, bedside table, bedside telephone) had high agreement.

The mental model category of **Disinfection** has potential as a precise heuristic because it represents the immediate practical implications of an object being inside or outside the patient zone. Indeed, the patient zone definition dictates indications for hand and object disinfection to prevent transmission of microorganisms between the patient and healthcare zones. However, participants in the current study faced challenges concretizing this deduction logic. In one notable case, a participant used the same logic (“it needs to be disinfected”) for allocating one item to inside and another to outside the patient zone. Here, participants seemed to correctly recognize the link between the patient zone concept and its IPC implications; however, gaps existed in the concretization of the concept. Another physician stated that HCP hands are inside the patient zone, “*Because hands have to be disinfected whenever you go to a patient.*” Although this allocation decision (inside) was incorrect, the practical implications that hands need to be disinfected before patient contact is correct. This suggests that participants intuitively understood the idea of zoning to prevent transmission, but the theoretical distinction of inside or outside patient zone seems to be disconnected from the practical implications.

Based on the findings of this study, the patient zone concept does not seem to fully resonate with HCPs as a practical approach to guide frontline IPC efforts. The various mental models held by participants in this study diverged from those of experts in important ways, leading to limited similarity and accuracy in patient zone allocations. Our analysis of these diverging mental models identified multiple sources of confusion, such as potentially misleading terminology around entering and exiting the patient zone and therefore promoting the misconception that the patient zone is a stable sector. Potentially attractive simplifying heuristics, such as equating the patient zone to the perimeter of the patient room, represent oversimplifications that do not adequately capture the nuances and complexities of the patient zone concept as conceived by experts. When considering the official WHO definition, the patient zone is defined as equivalent to “patient’s X immediate surroundings” [1]. This includes, “all inanimate surfaces that are touched by or in direct physical contact with the patient”, suggesting that the patient zone is indeed a geographical concept [1]. Indeed, the patient zone concept was conceived in the context of hand hygiene promotion as a scheme to facilitate and economize indications for hand hygiene. By defining which objects inside the patient zone are likely to be contaminated by the patient’s own flora, the intention was to remove the need for superfluous hand hygiene indications while frequently manipulating items in the patient’s direct environment during direct patient care. Indeed, the official WHO definition includes elements consistent with our participants’ **Patient contact** and **Sectors** mental models. Yet, these aspects alone do not seem to be selective enough for HCPs to clearly separate items from inside and outside the patient zone, particularly where mobile objects are concerned. The ambiguity surrounding attribution of mobile objects to inside or outside the patient zone may stem from the fact that it was originally conceived as a hand hygiene concept, but has evolved over time to also indicate need for object and environmental decontamination. Future research could therefore assess whether an alternate definition with adapted wording may be more selective and therefore perform better in an allocation task. In addition, it should be considered whether simply teaching the patient zone concept in theory is sufficient for ensuring the concept is understood and recalled during point of care activity. Yin et al., for example, demonstrated that introducing a physical patient zone demarcation was followed by improved hand hygiene compliance in a children’s intensive care unit [19]. While this study shows that environmental engineering interventions may be a promising avenue to guide IPC behaviors, such interventions must be designed with care to ensure that they promote accurate mental models. Whereas physical demarcations promote the idea of a delineated patient sector, other environmental restructuring, such as colored tagging of items, may be needed to promote understand of patient zone attribution as a fixed characteristic, particularly for mobile objects.

The results of our study may further help to explain the low hand hygiene adherence found in other observational studies, in particular after contact with objects in the patient zone [11, 12]. Such poor reported performance may, in part, reflect discrepancies between IPC expert observers’ and frontline healthcare providers’ mental models about how the patient zone is defined.

Some limitations to this study should be considered. First, our interviews were limited to participants from a single ward and experts from the infectious diseases department. This study would have to be extended to further care settings to allow more generalizable conclusions. Nonetheless, our qualitative analysis allowed us to derive rich insights even from a small number of participants. Second, we used a binary sorting method – this forced choice scenario (i.e. having to choose between inside or outside the patient zone) sometimes led to low agreement. Our qualitative analysis, however, allowed us to understand the subtleties and thought processes behind the binary sorting task that would have been overlooked by a purely quantitative analysis. Despite these limitations, the card-sorting task combined with semi-structured interviews allowed for an in-depth exploration of HCPs’ mental models necessary for the development of targeted interventions to improve frontline care providers’ understanding of the patient zone concept.

## Conclusion

Our analysis of HCPs’ mental models concerning the patient zone revealed significant ambiguity surrounding the patient zone concept, which led to low accuracy and similarity in item allocation. Participants’ understanding of the patient zone were based on simplifying heuristics, such as equating the patient zone to the perimeter of the patient room or to all items with patient contact. Such simplifications do not account for shared patient spaces or mobile objects used by multiple patients, and thus failed to accurately capture the intricacies of the patient zone concept as intended by experts. Mental models about which items belong to the patient zone also differed between HCPs, which represents a patient safety risk and increases the likelihood that HCPs could unknowingly transmit foreign microorganisms to the patient. For this reason, increasing HCP understanding of the patient zone and its patient safety implications should be an IPC priority.

To this aim, IPC initiatives to improve understanding of the patient zone should focus on establishing consistent mental models based on an exhaustive theoretical foundation directly linked to the practical implications. Our results provide first insights into which mental models are relevant for the assignment of items as inside or outside patient zone. This would enable a reassessment and if required, an adequate adaptation of the patient zone concept and thus, lead to an enhancement in infection prevention and patient safety.

## Data Availability

Not applicable.

## Abbreviations

HAI: healthcare associated infections
HCP: healthcare provider
IPC: infection prevention and control
